# Challenges Associated With the Use of Metal and Metal Oxide Nanoparticles as Antimicrobial Agents: A Review of Resistance Mechanisms and Environmental Implications

**DOI:** 10.1002/biot.70066

**Published:** 2025-07-25

**Authors:** Mpho Phehello Ngoepe, Stiaan Schoeman, Saartjie Roux

**Affiliations:** ^1^ DSI‐Mandela Nanomedicine Platform Nelson Mandela University Gqeberha Eastern Cape South Africa

**Keywords:** biogenic, environment, inorganic nanoparticle, metal resistance, resistance genes

## Abstract

The use of metal and metal oxide nanoparticles has been suggested as a means of combating antibiotic‐resistant bacteria (ARB). This is due to the ability of nanoparticles to target numerous sites inside the bacterial cell. Microbes can, however, develop a resistance to hazardous environments. Soil microorganisms have evolved resistance to specific metals in soil by employing alternative survival strategies, like those adopted against antibiotics. Because of this survival mechanism, bacteria have been able to develop defense mechanisms to deal with metallic nanoparticles. Resistance has evolved in human pathogens to therapies that use metallic nanoparticles, such as silver nanoparticles. Metallic nanoparticles and antibiotics have currently been proven to be ineffective against several infections. Due to these concerns, scientists are investigating whether nanoparticles might cause environmental harm and potentially breed microbes that are resistant to both inorganic and organic nanoparticles. The increased use of inorganic nanoparticles has thus been shown to result in contaminations in wastewater, facilitating horizontal gene transfer among bacterial populations. The resistance mechanism of metallic nanoparticles, role in antibiotic resistance, and a potential solution to the environment's toxicity from nanoparticles are all discussed in this review.

## Graphical Abstract and Lay Summary

Nanoparticles are efficient against biofilms and resistant bacteria, but prolonged exposure may result in resistance and gene transfer. The review discusses strategies like rotation therapy and phytoremediation, along with green synthesis procedures, to reduce these risks and make nanoparticles safer inside the body and environment.

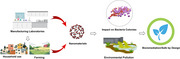

## 1 Introduction

**FIGURE 1:**
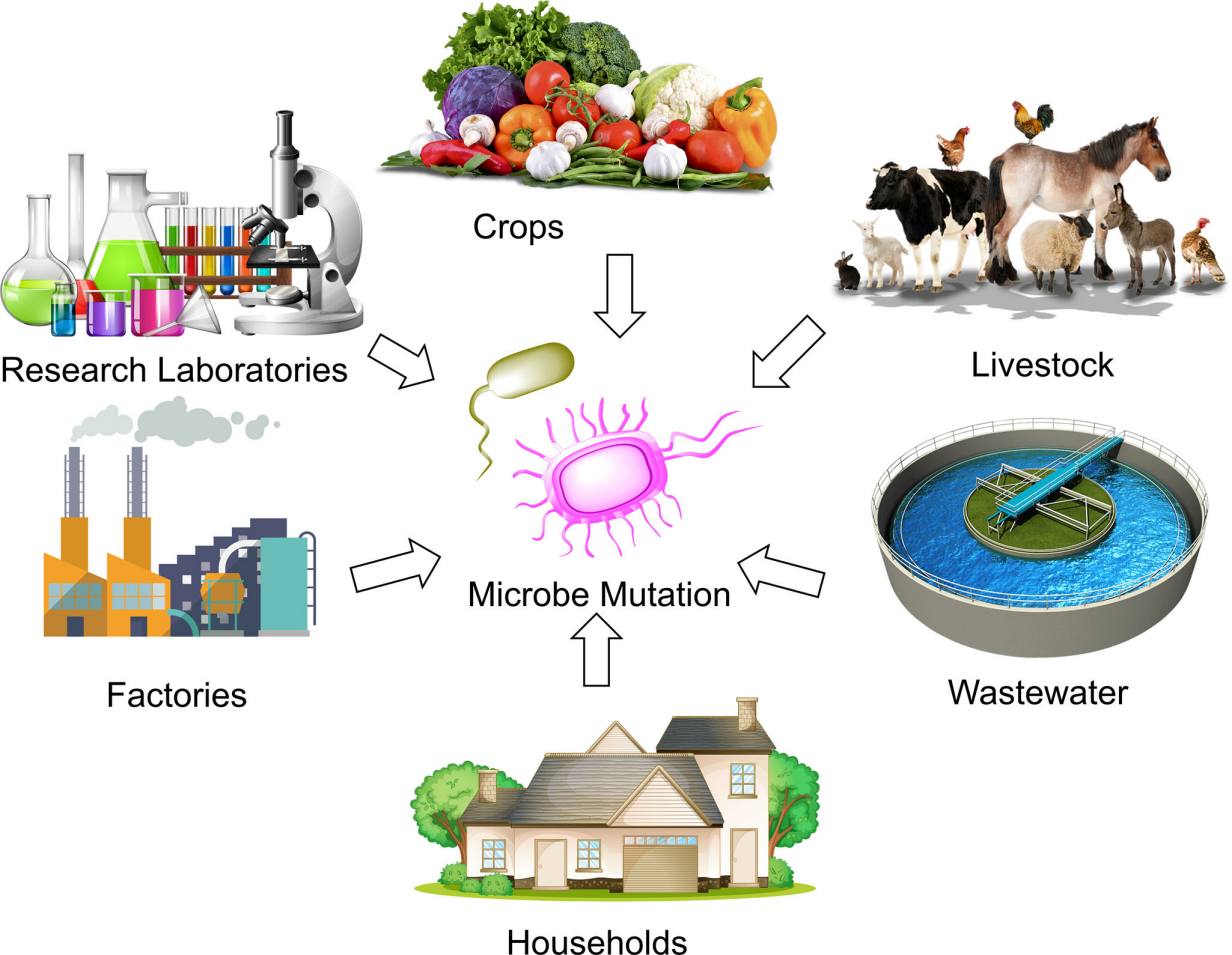
Pathways of nanoparticle environmental release and their contribution to microbial mutation and resistance development.

Globally, silicon dioxide (SiO₂), titanium dioxide (TiO₂), and zinc oxide (ZnO) are the top three nanomaterials manufactured, with annual production volumes of 5500, 3000, and 550 tonnes, respectively [1]. Nanoparticles found in consumer products pose environmental risks by accumulating in aquatic systems and harming algae, bacteria, and invertebrates [2]. Silver (Ag) and zinc (Zn) nanoparticles, which are zero‐valent metals, prevent the growth of bacteria by penetrating the cell membrane and generating reactive oxygen species (ROS) [3]. Due to physical, chemical, and biological changes in environmental systems, the bioavailability, and toxicity of the created nanomaterials can be altered [4]. During the processes of synthesis, distribution, application, and disposal, nanoparticles can enter the environment in several ways (Figure 1). Nanoparticles, introduced into the environment from various sources, can enter wastewater and agricultural systems, causing microbe mutation and the development of antimicrobial resistance genes (ARGs) and metal resistance genes. Antibiotic resistance genes (ARGs), which can spread between multiple bacterial species because of their high mobility (horizontal gene transfer), constitute an increasing threat to human health [5]. This may take place as a result of bacterial transformation (the direct absorption, incorporation, and expression of external DNA amongst closely related bacteria), transduction (the transfer of bacteriophage genes), and/or conjugation (conjugative plasmids transfer) [6]. Metal ions such as Cu^2+^, Ag^+^, Zn^2+^, and Cd^2+^, and metallic nanoparticles (MPNs) Al_2_O_3_, CuO, Ag, TiO_2,_ and ZnO promote the transfer of ARG via conjugation [7].

## 2 Overview of Metallic Nanoparticles and Antimicrobials

Antibiotics increasingly face resistance through microbial adaptation mechanisms like efflux pumps, enzymatic inactivation, and biofilms, all of which are worsened by overuse and low new drug development, thus undermining treatment efficacy. Metallic nanoparticles (MNPs), being an attractive alternative, can utilize multi‐target antimicrobial mechanisms that can evade traditional resistance mechanisms but minimize the potential for resistance development [8]. MNPs have superior antimicrobial actions that can be mechanistically described through reactive oxygen species (ROS) formation, membrane disruption, and targeted release of ions [9]. They have characteristics that allow them to overcome the weaknesses of common antibiotics, such as the ability to precisely act on resistant bacteria and fungi, while their hazards are much less than those of common agents [10]. MNPs offer green, broad‐spectrum antimicrobial alternatives with reduced toxicity. They can be potentially applied in biomedicine and agriculture, but their resistance mechanisms, such as oxidative stress adaptation and genetic mutation, are similar to conventional antibiotics [11]. Properly handling nanoparticle environmental implications, which include the development of resistance in natural environments and the long‐term accumulation effect, is thus essential.

## 3 Resistance Mechanisms

### 3.1 Mechanisms of Resistance to Nanoparticles

**FIGURE 2:**
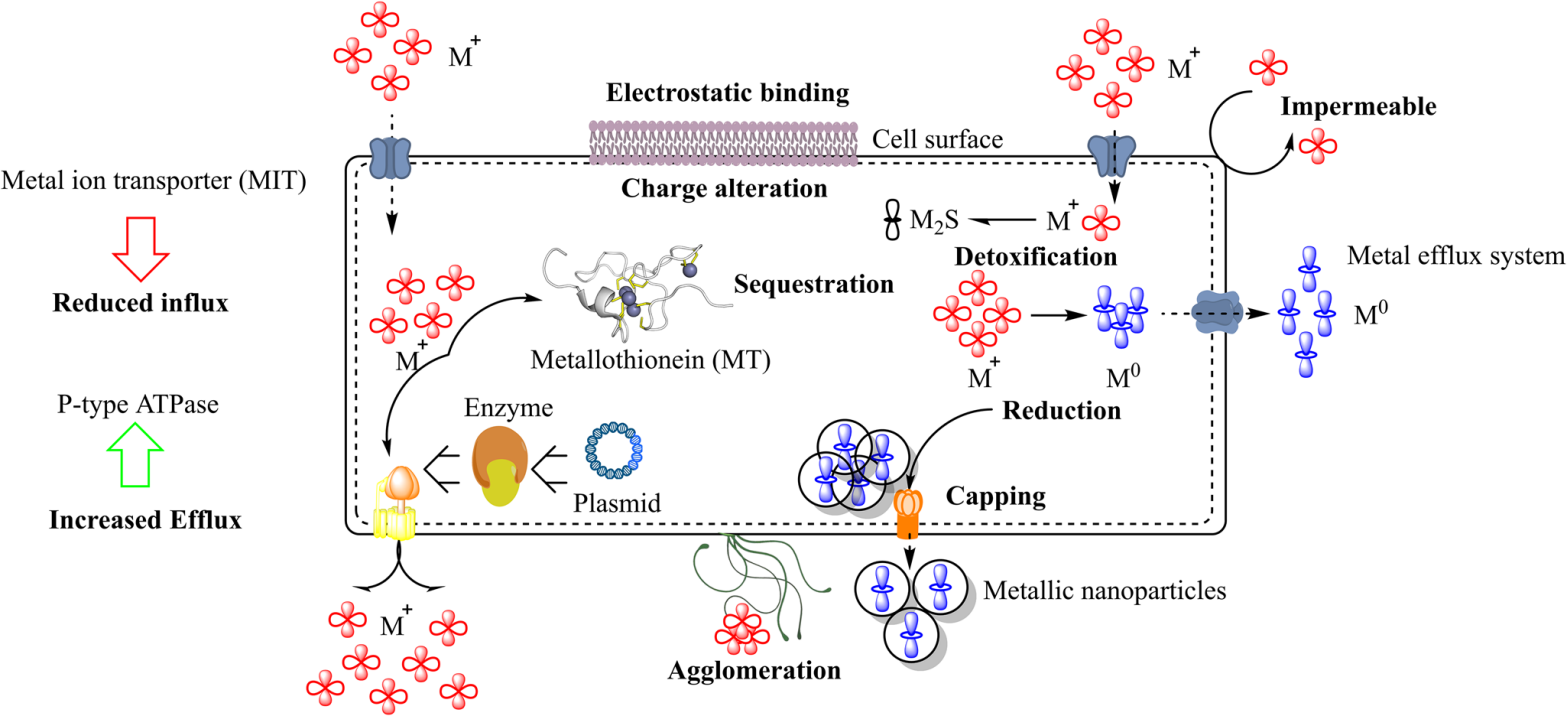
Bacterial strategies for detoxifying and resisting metal ions.

The therapeutic use of inorganic nanoparticles may be threatened by the irrational use of inorganic metals in human and animal medicine, analogous to the abuse of antibiotics [12]. Several mechanisms, including electrostatic repulsion, ion efflux pumps, the production of extracellular matrix for agglomeration and deactivation of NPs, the adaptation of biofilms that modify NPs' physicochemical properties, and mutations that reduce NPs' cellular entry, have already begun to emerge as resistance to various metal nanomaterial formulations (Figure 2) [13, 14, 15]. The mechanisms include drug inactivation, target receptor/site change, drug influx inhibition, drug efflux, and resistance gene (horizontal gene transfer), which have all been demonstrated with antibiotics [16]. One of the main causes has been noted as recurrent exposure to nanoparticle levels below the lethal threshold. It was found that exposure to low doses of ZnO at 0.2–1 and 10 mg/L led to an increase in the spread of ARGs such as sul1 and sul2 (sulfonamide resistance), tetA (tetracycline resistance), ermB (erythromycin resistance), qnrS (quinolone resistance), and tetW (tetracycline resistance), respectively [17]. Other mechanisms include upregulating antioxidant mechanisms, which enable ROS scavenging systems to overcome oxidative stress, downregulating cation‐selective porins genes to aid in the removal of metal ions from cells, and upregulating efflux pump genes to aid in the removal of metal ions from cells [18].

Microorganisms can serve as eco‐friendly bioreactors for the production of nanoparticles because they have a tolerance for toxicity as a result of biotransformation using metabolites such as proteins, chelators, and metallothioneins that have biological activity when used for medical purposes [19]. Iravani and Varma have studied various bacteria in bioreducing metal ions to create nanocrystals (Ag, Au, Ag–Au alloys, ZnS, Fe_3_O_4_, CdS, Pt, CdSe, and TiO_2_) of different shapes and sizes [20]. Metalloproteases, such as nitrate reductase, have been identified as the reducing agent in the bacterial‐mediated synthesis and stabilization of nanoparticles [21]. According to research on AgNPs as antimicrobials, the NPs' surface charge affects their ability to enter bacteria. By altering the charge of the bacteria's envelope and overexpressing pathogenic proteins, the envelope stress response decreases the bacteria's affinity for charged antimicrobials and results in the deactivation of NPs [22]. Porin downregulation limits the entry of smaller‐sized (10 nm) nanoparticles into the bacterial cell, whereas extracellular matrix production causes the aggregation and deactivation of nanoparticles larger than 10 nm [23]. Flagellin, an extracellular secreted sticky protein, has also been highlighted to cause nanoparticle aggregation [24]. Bacterial motility causes AgNPs resistance by moving bacteria away from the antimicrobial environment, although non‐motile strains are still susceptible to AgNPs inhibition [25]. Sulfidation has been identified as the main transformation and detoxifying step for silver nanoparticles in terms of chemical alteration (AgNPs) [26].

### 3.2 Nanoparticle‐Induced Mutagenesis

Metal ions such as Cd^2+^, Co^2+^, Cr^3+^, Hg^2+^, Mn^2+^, and Pb^2+^ have been found to have mutagenic effects, whereas Al_2_O_3_, Co_3_O_4_, TiO_2_, and ZnO NPs showed a negative response in a bacterial reverse mutation assay [7, 27]. Due to the nanoparticles' mechanism of action involving the release of ions, mutagenesis can occur [28]. Mono‐ and multi‐drug resistance genes can be found in bacteria exposed to nanoparticles, according to whole‐genome sequencing research of bacterial genes [29]. An investigation into the effects of Al_2_O_3_ and ZnO nanoparticles on bacteria has revealed that they put the organisms under ROS stress, which damages their DNA by oxidative damage and triggers an error‐prone SOS response that increases mutagenesis [29]. Beyond DNA damage repair, the SOS response plays important roles because it increases mutation rates, leading to genetic variety and adaptation, including antibiotic and metal resistance [30]. Proença‐Assunção et al. found that curcumin‐synthesized silver nanoparticles (Cur‐AgNPs) are mutagenic only in metabolic activation, and they can be indirect mutagens [31].

High frequencies of base‐substitution mutations and indicators of oxidative damage were detected in eukaryotic models by another research, suggesting that despite being potent antimicrobials, AgNPs pose a low but significant genotoxic risk to the eukaryotic genome [32]. Increased ROS, cellular stress, and damaged membranes have also been attributed to research on Ag, CuO, and ZnO‐based NPs/ions, all of which are essential for promoting transformation [33]. Specific genes involved in oxidative stress response pathways experience changes (upregulation) in their transcriptional and translational responses as a result of the metallic nanoparticles and their ions [34]. New ARGs, which are persistent molecules encoded in DNA, can form because of genetic mutations and, once they have established themselves, can transmit from parents to offspring or between nearby bacterial cells via horizontal gene transfer. Various genes have been extensively reviewed (Table 1) to play a role in metal resistance to both metal ions and nanoparticles [22]. Metal nanoparticle releases into the environment have the potential to trigger antibiotic resistance in bacteria through co‐regulation mechanisms, including transcriptional linking of heavy metal efflux genes and antibiotic resistance determinants [35]. High concentrations of cobalt, nickel, zinc, and cadmium in rivers can potentially drive co‐resistance through horizontal gene transfer and bacterial community composition shifts [36]. Heavy metal efflux genes and antibiotic resistance determinants are co‐regulated, thereby making bacteria antibiotic‐resistant. This is especially concerning in clinical settings where infections from antibiotic resistance are increasing. Heavy metals are also known to enhance horizontal gene transfer, which speeds up the spread of antibiotic resistance and complicates treatment decisions, potentially leading to public health crises.

**TABLE 1:** Primary genes involved in metal resistance.

Genes	Mechanism
CusS, SilP, SilE, SilF, SilCBA, AcrD	Efflux pump upregulation
CusS	*CusCFBA* efflux system
purl	Purine nucleotide biosynthesis
rpoB	RNA polymerase
copA	P‐type ATPase
PcoA, PcoC, PcoE	Periplasmic transport
tcrYAZB	Homeostasis
merRTPAGBDE	Detoxification system
IntI1, intI2, Tn916/1545, and ISCR1	Multidrug resistance mobile genetic elements (MGEs)
csgA	Biofilm formation

Various studies have shown the role of various genes in various microbial species. Bacteria develop resistance to silver AgNPs through mutations in regulatory pathways like CusS/CusR and OmpR, and the ArcA/ArcB pathway [37]. Black soldier fly larvae' guts copper resistance is mediated by upregulation of metal resistance genes (MRGs) as shown by metagenomic analysis [38]. *Escherichia coli* develops resistance to magnetite nanoparticles by cell size increase and RNA polymerase gene mutations, with extensive cross‐resistance to various metals and antibiotics [39]. Strain HNR adapts to ZnO nanoparticles through intracellular gene upregulation of protein repair, nitrogen metabolism, and ROS mitigation and extracellular modifications like enhanced exopolysaccharide (EPS) production and cell membrane modification [40]. Such genetic alterations are revealed by experimental evolution, selective sweep analysis, and polymorphism detection. While bacterial resistance to nanoparticles (e.g., AgNPs, ZnO) occurs via genetic mutation in systems like CusS/CusR or adaptive processes like EPS production and ROS detoxification, another study reveal that metal nanoparticles like ZnO can also promote antibiotic resistance by augmenting horizontal gene transfer, as attested by elevated transformation frequency in *E. coli* and elevated czcA gene copies in soil, pointing toward the dual role of nanoparticles in both combating and inadvertently spreading resistance [41].

## 4 Solutions to Counter Resistance

### 4.1 Mitigating Horizontal Gene Transfer via Nanoparticle Design

**FIGURE 3:**
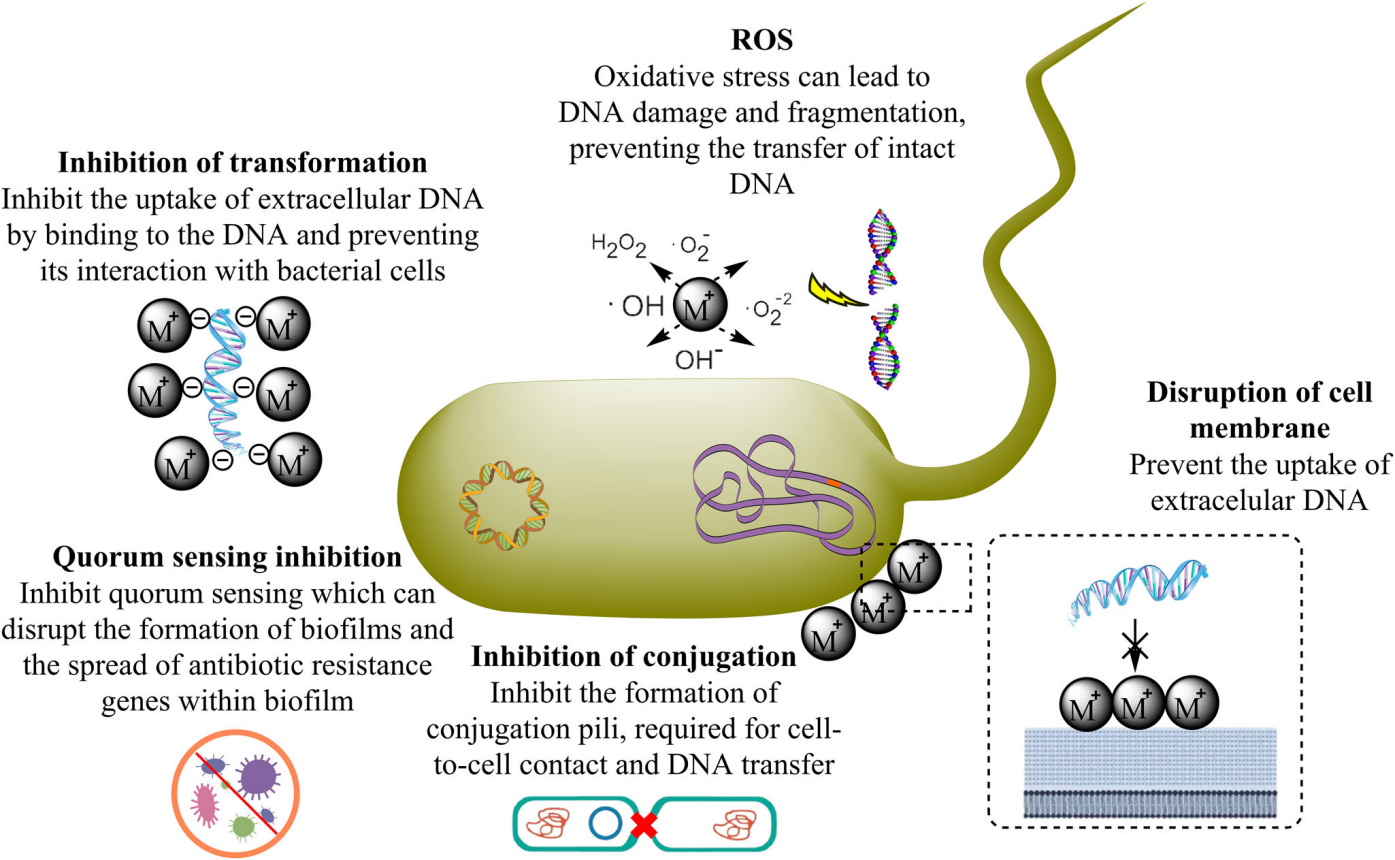
Proposed mechanisms by which metallic nanoparticles (MNPs) inhibit horizontal gene transfer and antimicrobial resistance in bacteria.

Utilizing genome studies, the absence of a genetic foundation for certain mechanisms of nanoparticle resistance can be leveraged to combat bacterial resistance. The research by Panáček et al. has demonstrated that the only way to overcome the resistance mechanism is by preventing the synthesis of flagellin using plant extract rather than further stabilizing AgNPs with surfactants or polymers [42]. The research conducted by Lu et al. demonstrated that in the absence of scavengers that prevent the excessive production of ROS, leading to increased cell membrane permeability, both Ag^+^ ions and AgNPs can facilitate the horizontal transfer of plasmid‐mediated antibiotic resistance genes (ARGs) [34]. At environmentally relevant and sub‐inhibitory concentrations (e.g., 1–100  µmol/L), both CuO nanoparticles (CuO NPs) and copper ions (Cu^2+^) were found to induce the conjugative transfer of multidrug resistance genes [43]. Different kinds of MNPs (Al_2_O_3_, AlO(OH), TiO_2_, SiO_2_, and Fe_2_O_3_ NPs) have been seen to promote (e.g., ZnO) or inhibit (e.g., TiO_2_) various gene transfer processes, impacting the spread of antibiotic resistance genes [44, 45]. Targeting the virulent factors that lead to nanoparticle resistance, the use of biogenic nanoparticles can overcome MNPs resistance. Metallic nanoparticles (MNPs) have been shown to inhibit both horizontal and lateral gene transfer by various mechanisms. The proposed mechanism (Figure 3) includes disruption of cell membranes, inhibition of conjugation, inhibition of transformation, ROS production, and quorum sensing inhibition [44, 46, 47, 48].

### 4.2 Dual Strategy Using MNPs and Natural Extracts

Metal oxide nanoparticles, such as titanium dioxide and zinc oxide, can effectively bind plasmids and inhibit lateral gene transfer (LGT) [49]. When metal oxide nanoparticles encounter plasmids, they can bind to the DNA and prevent it from being transferred between bacteria. This is because the binding of the nanoparticle to the plasmid can interfere with the plasmid's ability to interact with the bacterial cell and replicate. Fe_3_O_4_@MoS_2_ nanocomposite has also been shown to inhibit HGT by decreasing the permeability of bacterial cells against exogenous DNA, whereby genes involved in conjugative transfer were inhibited [50]. Phytochemicals have also been observed to have the ability to alter the expression of conjugation‐associated genes, inhibiting antibiotic resistance transfer [51]. The extract derived from *Myristica lowiana* exhibited a notable inhibitory effect on the conjugal transfer of the IncW plasmid R7K [52]. The IncW plasmid R7K contains genes conferring resistance to ampicillin, streptomycin, and spectinomycin [53]. Extracts from *Mikania micrantha* Kunth can significantly reduce ARGs abundance and decrease pathogen‐host interactions (PHIs) [54]. MNPs can inhibit horizontal and lateral gene transfer by various mechanisms, making them potential candidates for the development of antimicrobial agents that can prevent the spread of antibiotic resistance. However, further research is needed to optimize the use of MNPs for this purpose and to evaluate their safety and efficacy in vivo.

In a recent research work, ciprofloxacin, cefixime, bacitracin, tetracycline, fluconazole, and metronidazole were employed, where biogenic AgNPs showed synergism (enhanced antibiotic activity) against *Pseudomonas aeruginosa* and *E. coli* but antagonism (lost activity) with bacitracin/ciprofloxacin against *Staphylococcus aureus*, whereas co‐administration of AgNPs with fluconazole/metronidazole showed enhanced antifungal activity against *Candida albicans* [55]. Another study of biosynthesized AgNPs using *Acinetobacter baumannii*, which, when combined with antibiotics like ceftriaxone, exhibited synergistic and partial synergistic activity (reducing antibiotic doses significantly) against carbapenemase‐producing Gram‐negative bacilli (CPGB), showing promise as a novel nanomedicine approach to combat multidrug‐resistant infections [56].A study on a cocktail of polyol‐coated CuO, ZnO, and CuZnNPs showed a high level of synergy with antibiotics like ciprofloxacin and meropenem against multidrug‐resistant (MDR) *Pseudomonas aeruginosa* by inhibiting efflux pumps, enhancing intracellular drug deposition, and re‐establishing the effectiveness of antibiotics at sub‐MIC concentrations [57]. A mixture of silver nanoparticles and bacteriophage ZCSE2 has been shown to significantly increase antibacterial activity against Salmonella, with a potential dual‐agent strategy for the treatment of antibiotic‐resistant disease [58]. These combinations optimize antimicrobial activity, evades resistance mechanisms, and reduces dose, but pairing needs to be optimal to prevent intermittent antagonism.

## 5 Environmental Impact

### 5.1 Antibiotic Resistance Genes (ARGs) Sensors and Removal

The introduction of quantitative polymerase chain reaction (qPCR) technologies has made it possible to quantify ARGs and conduct genomic analysis [59]. The application of end‐point PCR is limited by the need for well‐designed primers and prior genetic information. Additionally, the accuracy of detecting ARGs through this method may be hindered by constraints such as limited throughput, amplification bias, and the potential for misleading outcomes, including false‐negative or false‐positive results [60]. It has been demonstrated that a novel few‐layered, two‐dimensional graphdiyne nanosheet (GDY NS) may identify *Mycobacterium tuberculosis* (MTb) and its drug‐resistant genes [61]. Once modified with recognition components like antibodies, phages, or aptamers, surface plasmon resonance (SPR)‐capable plasmonic nanoparticles like gold and silver NPs are frequently utilized for bacterial detection. Within 3 h, a study on an AuNP‐based lateral flow assay demonstrated its ability to distinguish between a particular mutation and the wild type, in addition to identifying MTb [62]. DNA has a molecular recognition function and has been used to synthesize nanomaterials (especially Au) as a capping agent for the detection of bacteria, miRNA, metal ions, proteins, nucleic acids, and drugs [63]. Antimicrobial resistance genes have been detected using aptamer‐Fe_3_O_4_@Au magnetic nanoparticles (AuMNPs) [64].

Historically, chlorination has been the primary method of disinfection used in drinking water treatment due to its cost‐effectiveness and effectiveness in oxidizing and damaging the cell surface, nucleic acids, and DNA structure of microorganisms [65]. The selection of chlorine‐resistant bacteria (CRB) has also been accelerated by chlorine disinfectants in drinking water; ultraviolet (UV) irradiation has been proposed as a successful CRB control technology [66]. In a study conducted by Destiani and Templeton, it was shown that the combination of ultraviolet (UV) treatment and chlorine addition did not achieve complete disinfection of ARGs in drinking water [67]. Numerous advanced nanomaterials have been suggested as catalysts for the eradication of ARB and the elimination of ARGs [60]. To enhance the effectiveness of removing ARGs to a greater extent, the implementation of supplementary technologies such as advanced oxidation processes (AOPs) is recommended [68]. AOPs can damage the cell surface and DNA structure through free radical (i.e., •OH, SO_4_^•−^, and O_2_^•−^) reactions [69]. The free radical can be generated through the Fenton process (H_2_O_2_ and Fe^2+^), UV/H_2_O_2_, ultrasonic, ozonation (O_3_), photocatalysis (e.g., TiO_2_), and sulfate radical‐based AOPs (SR‐AOPs) [60]. The functionalization of the nanoparticles with antibiotics, natural substances, or chaotropic salts with a positive charge that may draw a sufficient number of harmful ions is necessary to eliminate ARGs (DNA conveying ARGs) [70].

Apart from free radical damage, the study on ZnO, Al_2_O_3_, and TiO_2_ nanoparticles at concentrations of less than 50 mg/L was shown to inhibit lateral gene transfer (LGT) due to antibiotic resistance plasmid DNA phosphate groups and bases interaction with nanoparticles forming aggregates that prevented entry into bacterial cells. Two specific regions on a DNA molecule where metal cations can directly or indirectly interact are the negatively charged phosphate backbone and the distinct high electron density sites represented by the nitrogen (N) and oxygen (O) atoms of the nucleobases [71]. These interactions range from intercalation (transition metals), irreversible covalent binding (divalent alkali earth metals), and groove association (e.g., alkali metals) [72]. Thus, nano‐zerovalent iron (nZVI) [Fe^0^] has been utilized to remove pollutants through adsorption, flocculation, and oxidation due to its strong adsorption property and redox activity that leads to DNA damage [73]. Graphene oxide (GO), which can adsorb different pollutants and biomolecular interactions with RNA and DNA, was observed to possess high ARGs efficiency [74].

### 5.2 Nanoparticle Marine Environment Interaction

Metal speciation is a complex process that occurs when MNPs are exposed to a different environment. It is influenced by factors such as organic matter, concentration of ligands, pH levels, and ionic strength [4]. The study confirmed that Cd^2^⁺ and nano Fe₂O₃ exposure synergistically improved the transfer of antibiotic resistance genes among bacteria, especially to human pathogens [75]. This highlights the possible risk of nanoparticle contamination in accelerating resistance diffusion and requiring a stricter evaluation of nanoparticle–heavy metal interactions in resistance management programs. Interaction with natural organic matter (NOM) can lead to adsorption and desorption (electrostatic interactions) and chemical reactions such as reduction and oxidation, depending on the surface properties [76]. In contrast, the presence of NOM can promote increased stability of MNPs (steric repulsion) through surface oxidation and sulfidation, resulting in a decrease in the overall uptake of MNPs by organisms [77]. Wang et al. [78] provide an overview of the key biological impact of NOM on nanomaterials in aquatic environments. They focus on how NOM adsorbs onto NMs, forming an eco‐corona, which affects nanoparticle stability, aggregation, and surface properties, with an eye to their potential biological impacts on aquatic organisms (Table 2)[78].

**TABLE 2:** Mechanisms involved in the interaction of nanomaterials with natural organic matter (NOM).

Driving force	Mechanism
van der Waals interactions	Non‐specific, attractive forces between NOM molecules and the surface of nanomaterials.
Hydrogen bonding	Interaction between hydroxyl, amino, or other functional groups on NOM and nanomaterials.
Hydrophobic interactions	Hydrophobic domains in NOM will interact with hydrophobic surfaces of nanomaterials, especially in high ionic strength solutions (e.g., seawater).
Electrostatic interactions	Attraction or repulsion between charged NOM (e.g., humic acids) and nanomaterials by pH and ionic strength.
Covalent bonding	Possible covalent bond formation by chemical reaction, especially during surface modification or under reactive conditions.
Bridging mechanisms	Organic molecules or NOM as bridges, causing aggregation or stabilization of nanomaterials through multidentate interactions

These NOM‐induced alterations may influence nanomaterials' environmental fate and bioaccumulation ability, as evidenced in the case of marine molluscs during seawater bioaccumulation of MNPs such as Ti, Cu, Zn, and Ag in gills and digestive gland but at non‐hazardous levels for application in human consumption based on the study of estimated daily intake (EDI) [79]. The growth of algal cells was found to be hindered, and the chlorophyll content of *Chlorella vulgaris* was reduced when Ni^2+^ was released from nickel oxide nanoparticles (nNiO) due to the conglomerate of nNiO in seawater [80]. The release of Zn^2+^ from zinc oxide nanoparticles (ZnO) present in commonly used sunscreens can induce oxidative stress, potentially threatening marine life [81]. Under marine conditions, the noxious impact of Ag^+^ was found to be lessened owing to the presence of a substantial concentration of chloride ions (Cl^−^), which resulted in the oxidative dissolution of AgNPs and the formation of Ag/AgCl‐NPs and AgCl*x*(*^x^* ^−1^) compounds [82].

MNPs undergo degradation when exposed to the lysosome's cysteine‐mediated reductive environment, resulting in the release of large amounts of ions capable of disrupting the normal balance of metal ions and generating excessive reactive oxygen species (ROS). Monitoring the production of ROS during exposure to MNPs can serve as a biomarker for assessing toxicity [83]. Elevated levels of metallothioneins are recognized as an established biomarker for MNP‐induced toxicity. Exposure to ROS can also cause DNA damage. The review by Roma et al. evaluated the effects of MNPs effects on marine fauna, and it was highlighted that concentrations above 100 µg/L, ligands (coatings), size (penetrate an organism), particle behavior (photoactivation, dissolution, and aggregation/agglomerate), and time of exposure (longer periods) play a role in toxicity [84]. When exposed to nano‐sized titanium dioxide (nTiO_2_) in natural oligotrophic seawater, the microbial population of the dominant marine cyanobacterium initially declined [85]. After 72 h, the nanoparticles agglomerated and settled out of the solution, leading to the recovery of the cultures [85]. This indicates that transient physical effects (entrapment by aggregates) are accountable for the short‐term decrease in the microbial population.

Several marine plants (*Rhizophora mucronate* [mangrove]), algae (e.g., seaweeds), cyanobacteria (e.g., *Oscillatoria* sp.), bacteria (e.g., *Streptomyces* sp.), fungi (e.g., *Aspergillus* sp.), yeasts (e.g., *Saccharomyces* sp.), invertebrates (e.g., Polychaetes), animals (e.g., *Nemopilema nomurai* [giant jellyfish], seahorse), sponges (e.g., *Amphimedon* sp.) have been used for bioreduction, capping, and stabilization of various MNPs due to diverse classes of bioactive secondary metabolites [86]. Marine organisms possess the capability to thrive in harsh environments, which has led to the production of diverse chemical constituents that differ significantly from those found in their terrestrial counterparts [87]. MNPs synthesized by marine organisms frequently showcase distinctive characteristics, including antibacterial, anticancer, cytotoxic, antioxidant, antifungal, and catalytic properties [88]. Proteins and polysaccharides are the organic components that are commonly utilized by marine organisms in the synthesis of MNPs [89]. Proteins can bind with nanoparticles through their free amine groups or cysteine residues, resulting in the formation of a protein coating around MNPs that prevents aggregation and stabilizes the medium [90]. Toxicity studies on MNPs have limitations, such as higher concentrations of chronic exposure and variations in physical and chemical properties between natural water and laboratory culture medium [91]. Thus, this highlights the need for further investigation to better understand the real‐world implications and potential risks associated with MNPs.

## 6 Removal Strategies

### 6.1 Nanoparticle Removal From the Environment

**FIGURE 4:**
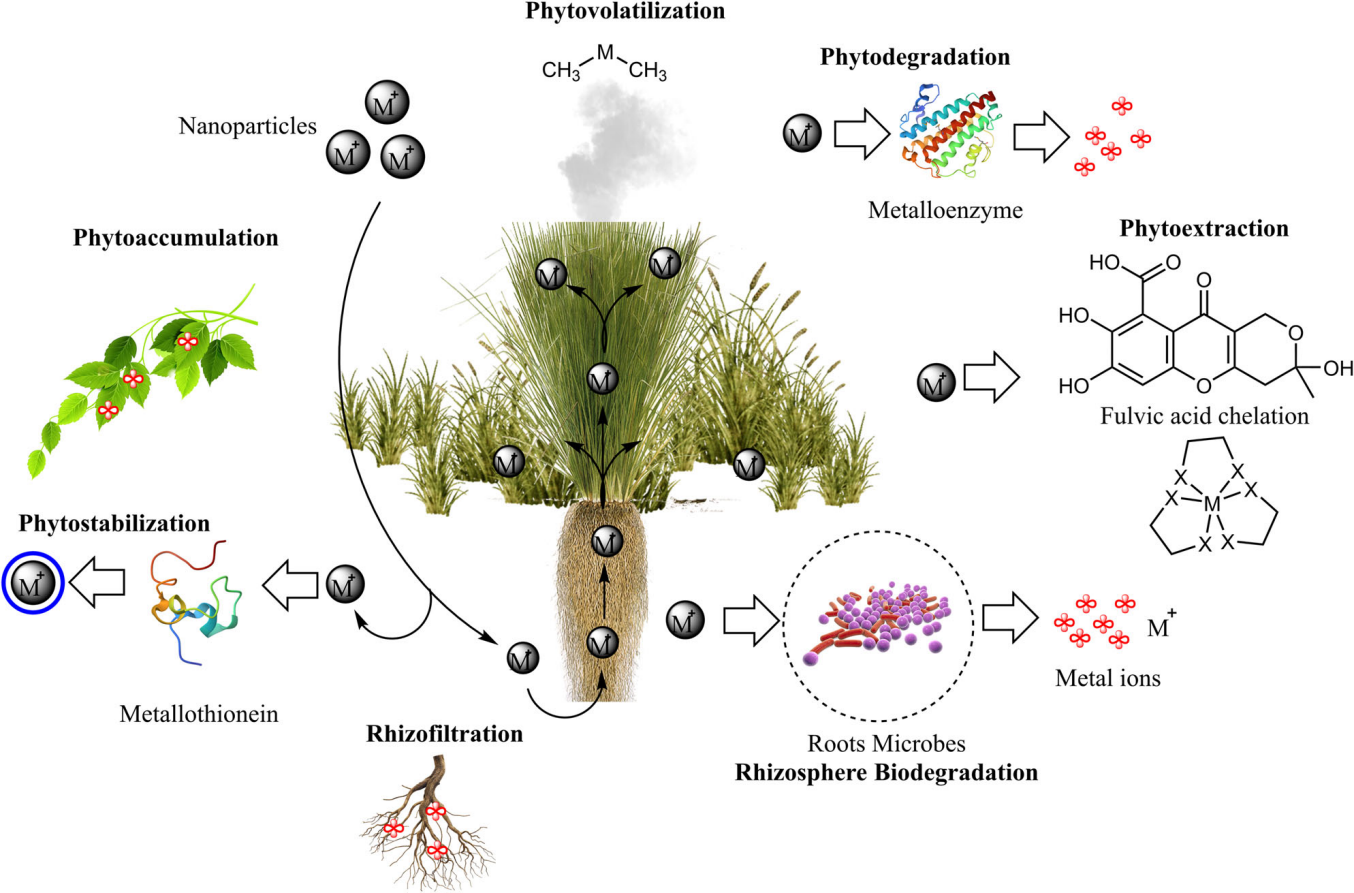
Plant‐assisted remediation strategies for removing metallic nanoparticles (MNPs) from the environment.

Chemical and physical methods can be used to remove inorganic nanoparticles from soil and water in several ways. The use of chemical absorbents such as mesoporous silica, condensation, evaporation, and magnetic field/materials has limits in comparison to phytoremediation, which is an inexpensive, environmentally benign, and simple process [92, 93]. The use of plants to remove pollutants from soil, air, and water resources is known as phytoremediation (Figure 4). *Pistia stratiotes* (water lettuce) was found to eliminate AgNPs produced utilizing leaf extract from *Muntingia calabura* (Jamaica Cherry) species [94]. Aquatic plants (e.g., duckweeds, waterweeds, and macrophytes) have been observed to be able to remove 30%–100% of AgNPs (≤ 0.02 ppm) from water without exhibiting any toxic effects [95]. The study by Song et al. has shown that the plant species *Suaeda glauca* (seepweed) could remove TiO_2_ and ZnO nanoparticles from nanomaterial‐contaminated soil [96]. The nanoparticles have been observed to play a role in phytoremediation of heavy metals such as aluminum (Al), Arsenic (As), lead (Pb), mercury (Hg), cadmium (Cd), nickel (Ni), chromium (Cr), iron (Fe), copper (Cu), selenium (Se), and zinc (Zn) [97].

The plant, *Chrysopogon zizanioides* (vetiver) is a hyperaccumulator of metals; its roots can hold significant amounts of heavy metals such as arsenic (As), cadmium (Cd), chromium (Cr), lead (Pb), copper (Cu), nickel (Ni), selenium (Se), zinc (Zn) and mercury (Hg). As a result, it has been successfully used in the field of environmental protection [98]. The metals are removed via various mechanisms as follows: rhizosphere biodegradation, rhizofiltration, phyto‐stabilization, phyto‐accumulation, phyto‐volatilization, phytoextraction, and phyto‐degradation [99]. Nanoparticles‐assisted phytoremediation is a process whereby nanoparticles can facilitate plant phytoremediation through interaction with heavy metals by adsorption/redox reactions and stimulate plant growth (in combination with rhizospheric microbes and fungi) [100, 101]. Thus, plants can be used to remove the synthesized nanomaterials' environmental contamination apart from being used during nanoparticle synthesis. In the research conducted by Noman et al., a copper‐resistant strain of *Shigella flexneri* SNT22 was utilized to successfully synthesize copper nanoparticles (CuNPs) [102]. The application of these CuNPs resulted in a 44.4% increase in wheat plant length, a 28.26% boost in shoot dry weight, and a reduction of acropetal cadmium translocation by 49.62% in soil contaminated with metals. Thus, aquatic macrophytes show remarkable promise for NP removal from aqueous conditions [103].

## 7 Challenges and Future Perspectives

The translation of MNPs as antimicrobial agents into the clinic is significantly hampered by issues related to their long‐term biocompatibility, toxicity, and possible environmental effects, which present regulatory hurdles and limited adoption [104]. Metal nanoparticles face significant financial issues in the formulation and clinical studies process, the lack of regulatory pathways, as well as biocompatibility issues in the long‐term in vivo manner, thus complicating their use in medicinal applications compared to biodegradable polymeric or liposomal nanoparticles [105]. Nanotechnology‐Enabled Health Products (NHPs) are regulated worldwide according to established frameworks for medical products or medical devices [106]. The United States and European Union are leading examples, with NHPs being regulated respectively by the U.S. Food and Drug Administration (FDA) and European Medicines Agency (EMA) through established routes [106]. Other regions, however, have non‐harmonized nano‐specific regulations (e.g., varying definitions of nanomaterials, validated and standardized characterization methods, safety, and toxicity assessment strategies), resulting in issues such as inconsistent classification, sparse guidance, and changing environmental and safety factors.

Nanomaterials (NMs) are used in various applications like augmenting diagnosis, drug delivery, vaccines, purification of water pollutants, replacing hazardous chemicals used in crops, reducing water pollution in solar cells, and developing quality, reliable, sustainable, and resilient infrastructure in industries like coating and painting [107]. However, due to unique properties (e.g., size), nanomaterials cannot be discarded using conventional methods. Activities such as recycling and reusing, waste treatment, and degradation studies have been proposed. Recycling and reusing involve effective management and separation of nanomaterials. Treatment processes of waste should be harmless and effective to reduce environmental and health issues. Degradability studies help identify nanomaterials' persistence and long‐term effects, contributing to regulations and guidance for health and environmental protection. These challenges will have to be overcome by thorough safety evaluation, better characterization methods, and countermeasures against environmental hazards to ensure the effective and safe deployment of MNPs in antimicrobial applications.

## 8 Conclusion

Biogenic MNPs have emerged as potential strategies for combating the rapid proliferation of antibiotic‐resistant microorganisms and controlling infections. These nanoparticles possess adjustable properties that make them effective antibacterial agents against biofilms and antibiotic‐resistant bacteria. However, recent studies have revealed that prolonged exposure to metallic nanoparticles may contribute to the development of resistance mechanisms and facilitate the spread of antibiotic‐resistant genes. Activation of competence and SOS response‐related genes by nanoparticles and ions can promote the natural transformation of antibiotic‐resistance genes, potentially promoting the emergence of antimicrobial resistance. It is crucial to exercise caution when using nanoparticles for antimicrobial purposes. One approach to limit the development of resistance is rotation therapy, which involves alternating the use of different types of metallic nanoparticles cyclically. Additionally, plants can be utilized for phytoremediation to remove nanoparticle contamination, as well as act as bioreactors for nanoparticle synthesis to combat antimicrobial resistance. Employing green synthesis methods can help mitigate the toxic properties of metallic nanoparticles by enhancing their behavior and biocompatibility.

## Author Contributions

**Mpho Phehello Ngoepe**: writing – review and editing, writing — original draft, visualization, methodology, investigation, conceptualization. **Stiaan Schoeman**: writing — review and editing. **Saartjie Roux**: Writing — review and editing, funding acquisition.

## Conflicts of Interest

The authors declare no conflicts of interest.

## Data Availability

No data were used for the research described in the article.
